# Performance comparison of RGB and multispectral vegetation indices based on machine learning for estimating *Hopea hainanensis* SPAD values under different shade conditions

**DOI:** 10.3389/fpls.2022.928953

**Published:** 2022-07-22

**Authors:** Ying Yuan, Xuefeng Wang, Mengmeng Shi, Peng Wang

**Affiliations:** ^1^Institute of Forest Resource Information Techniques, Chinese Academy of Forestry, Beijing, China; ^2^Key Laboratory of Forest Management and Growth Modelling, National Forestry and Grassland Administration, Beijing, China

**Keywords:** *Hopea hainanensis*, chlorophyll, vegetation indices, machine learning, shade

## Abstract

Reasonable cultivation is an important part of the protection work of endangered species. The timely and nondestructive monitoring of chlorophyll can provide a basis for the accurate management and intelligent development of cultivation. The image analysis method has been applied in the nutrient estimation of many economic crops, but information on endangered tree species is seldom reported. Moreover, shade control, as the common seedling management measure, has a significant impact on chlorophyll, but shade levels are rarely discussed in chlorophyll estimation and are used as variables to improve model accuracy. In this study, 2-year-old seedlings of tropical and endangered *Hopea hainanensis* were taken as the research object, and the SPAD value was used to represent the relative chlorophyll content. Based on the performance comparison of RGB and multispectral (MS) images using different algorithms, a low-cost SPAD estimation method combined with a machine learning algorithm that is adaptable to different shade conditions was proposed. The SPAD values changed significantly at different shade levels (*p* < 0.01), and 50% shade in the orthographic direction was conducive to chlorophyll accumulation in seedling leaves. The coefficient of determination (*R*^2^), root mean square error (RMSE), and average absolute percent error (MAPE) were used as indicators, and the models with dummy variables or random effects of shade greatly improved the goodness of fit, allowing better adaption to monitoring under different shade conditions. Most of the RGB and MS vegetation indices (VIs) were significantly correlated with the SPAD values, but some VIs exhibited multicollinearity (variance inflation factor (VIF) > 10). Among RGB VIs, RGRI had the strongest correlation, but multiple VIs filtered by the Lasso algorithm had a stronger ability to interpret the SPAD data, and there was no multicollinearity (VIF < 10). A comparison of the use of multiple VIs to estimate SPAD indicated that Random forest (RF) had the highest fitting ability, followed by Support vector regression (SVR), linear mixed effect model (LMM), and ordinary least squares regression (OLR). In addition, the performance of MS VIs was superior to that of RGB VIs. The *R*^2^ of the optimal model reached 0.9389 for the modeling samples and 0.8013 for the test samples. These findings reinforce the effectiveness of using VIs to estimate the SPAD value of *H. hainanensis* under different shade conditions based on machine learning and provide a reference for the selection of image data sources.

## Introduction

The protection and artificial cultivation of endangered plants have always been a concern worldwide and are of great significance to the maintenance of ecological diversity (Timyan and Reep, 1994; Wang et al., 2021). However, the practical problems existing in protection and cultivation still pose huge challenges to the extensive cultivation of endangered plants. For instance, we have insufficient knowledge of the changes in physiological characteristics in the process of plant growth and cultivation, and we cannot obtain quantitative analysis results through a large number of destructive chemical experiments, so it is difficult to create a more suitable growth environment for endangered plants. *Hopea hainanensis* Merr. et Chun is a tropical endangered tree species mainly distributed in Hainan, China, and Nghe An, Vietnam (Song et al., 2020). Due to the slow growth rate and the sharp reduction in the number of these trees caused by human interference and destruction to the living environment, *H. hainanensis* has been rated as “endangered” (EN) by IUCN. Wild *H. hainanensis* is often grown in tropical rainforests and is shade tolerant, but corresponding investigations and research are still lacking.

Chlorophyll is a compound that directly affects photosynthesis and is also an important indicator of plant growth. Some studies determined the most suitable light conditions for plant growth by analyzing changes in the chlorophyll content under different shade conditions (Senevirathna et al., 2003; Dai et al., 2009). Rapid and timely growth monitoring can be realized through rapid and nondestructive estimation of chlorophyll, thereby leading to more extensive artificial cultivation of endangered tree species, such as *H. hainanensis*. A portable chlorophyll analyzer can be used for the nondestructive determination of the chlorophyll content. The SPAD values measured by such an instrument have been shown to be effective in reflecting the chlorophyll content. Azaruddin et al. (2006) suggested that SPAD meter was potentially useful as an alternative to assess leaf chlorophyll of *Hopea odorata*. However, in practical applications, it is difficult to meet the data needs of large samples because of the manual nature of these measurements. Thanks to the development of automation, artificial intelligence and Internet of Things, the acquisition and transmission of image data is much easier than manual measurement. Image-based estimation method can be used as the basis of remote monitoring, even real-time monitoring. Reflection changes in nutrients and water in plants assessed by spectral information make the image-based estimation method feasible. Hyperspectral imaging technology can capture the rich spectral information of plants. In previous studies, the chlorophyll content was retrieved with high accuracy by selecting spectral indices highly correlated with the chlorophyll content from hyperspectral data to build models (Wang et al., 2019). However, it cannot be ignored that a large amount of spectral information also brings data redundancy and high-cost problems, which create difficulties for practical application. RGB and multispectral (MS) images deserve to be considered low-cost alternatives.

Agarwal and Gupta (2018) analyzed the reflectance information in the RGB images of leaves by using multivariate data analysis tools, including principal component analysis and agglomerative hierarchical clustering analysis, to distinguish between spinach seedlings with high and low chlorophyll contents. Liang et al. (2017) chose the exponential function model to estimate the chlorophyll content of *Arabidopsis* grown under different sugar nitrogen ratios with the red, green, and blue values of RGB images of leaves and fitted the model parameters with the physically measured value of the chlorophyll content. To avoid the difficulty of using standard chemical procedures for chlorophyll measurement, Hassanijalilian et al. (2020) took RGB images of soybeans under field conditions with smartphones and selected the VIs with the best correlation with the SPAD meter readings to build an estimation model for the estimated chlorophyll content of soybeans. Qi et al. (2021) calculated eight VIs through MS images obtained on a UAV platform and studied the ability of MS VIs to estimate the chlorophyll content of two types of peanuts, Yanghua 1 and Yueyou 45, under different planting densities. The results showed that this method could quickly obtain information on the chlorophyll content in the field and could infer the most suitable crop type and planting density for local planting conditions. These studies showed the performance of RGB and MS images in nutrient estimation but did not give a specific opinion on the selection of image data sources through comparison. In recent years, machine learning has developed rapidly and has been gradually introduced into the field of plant science (Xie et al., 2022).

Shade has obvious effects on the photosynthetic performance of plants and is reflected in the chlorophyll content (Senevirathna et al., 2003). A very convenient, common, and low-cost method to increase the growth of seedlings is the use of shade nets or other tools to control shade in the seedling cultivation base. Experiments conducted on *H. hainanensis* seedlings showed that the growth condition of the seedlings under shade was better than that under full light (Yang and Chen, 2017), but regrettably, quantitative mathematical analysis results have not been published. In previous studies on nutrient estimation, light conditions were considered in the experimental design, but differences in plant performance at different shade levels have rarely been discussed in model construction. It is conducive to wide application at multiple levels, but there is a loss of model accuracy.

Based on the above considerations, the main purpose of this paper is to propose a nondestructive SPAD estimation method for *H. hainanensis* seedlings under different shade conditions and to determine the most suitable image data source. We set up four shade levels (0%, 25%, 50%, and 75%) in the experiment, measured the SPAD value representing the chlorophyll content and obtained the RGB and MS images of *H. hainanensis* seedlings. The Lasso algorithm was used to screen VIs, and the performances of RGB and MS VIs were compared based on ordinary least squares regression (OLR), a linear mixed effect model (LMM), a random forest (RF) model, and support vector regression (SVR). The specific objectives were as follows: (i) To determine the most suitable shade conditions for the growth of *H. hainanensis* seedlings through quantitative analysis of SPAD differences under four shade levels. (ii) To select the most suitable RGB and MS VIs and eliminate multicollinearity by the Lasso algorithm. (iii) To improve the application ability of the model at multiple shade levels by introducing random effects or dummy variables and find the optimal modeling algorithm. (iv) To propose suggestions for the selection of image data sources.

## Materials and methods

### Study area

**Figure 1:**
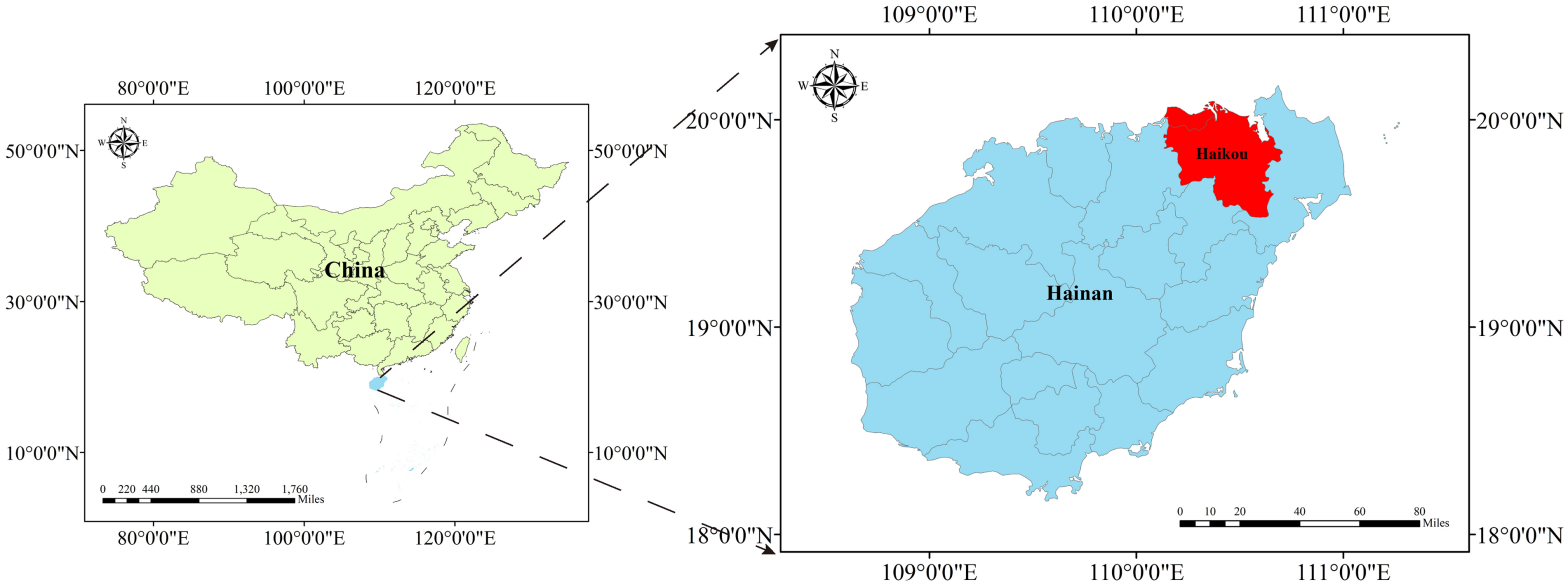
Location of study area.

Field trials were carried out in southern China, in Haikou (110°28′08″E, 19°52′25″N), Hainan, which has a tropical monsoon climate (Figure 1). The study area planted with 200 2-year-old *H. hainanensis* seedlings was divided into four parts according to shade conditions. The four shade levels were controlled as 0%, 25%, 50%, and 75% shade by shade nets erected in an orthographic direction. There were 50 repeats for each level and 1.2 m between plants in each row. In the experimental area, all field management measures were consistent except for the shade conditions. During the trial period, the average daily maximum temperature was 34°C, the average daily minimum temperature was 26°C, and the total rainfall was 706 mm.

### SPAD and image data collection

#### Field measurements

In August 2021, the key period for the growth of *H. hainanensis* seedlings, the SPAD values were measured by a portable plant nutrient meter (TYS-4N). To improve the representativeness of the samples, three leaves of each seedling were randomly selected for SPAD measurements, and the average values were calculated. The average height of plants was 38.9 cm and the average crown width was 20.6 cm. The canopy images of the seedlings were captured in a downward direction by cameras ~2 m above the ground. The distance from the camera to the plant was sufficient. The RGB images were captured by a digital camera (Canon EOS 4000D) in semiautomatic aperture priority shooting mode. The MS images were acquired by an MS camera (MicaSense Edge 3™) with five narrowband spectral sensors. The specific parameters of the spectral sensors are shown in Table 1.

**Table 1:** Specific parameters of the spectral sensor.

Spectral band	Center wavelength (nm)	Bandwidth FWHM (nm)
Blue	475	20
Green	460	20
Red	668	10
Near IR	840	40
Red Edge	717	10

#### Image processing

Through visual interpretation, we extracted regions of interest from the original image that clearly highlighted individual seedlings. In this way, the calculation cost for subsequent image analysis was reduced. A study reported that removing background disturbance can prevent the compound influence of the background (e.g., flood water, bare soil, algae, etc.) on spectral information to improve the robustness of VIs (Wang et al., 2022). In this study, VIs were used in combination with the traditional threshold segmentation method to remove the background in the seedling canopy images.

**Figure 2:**
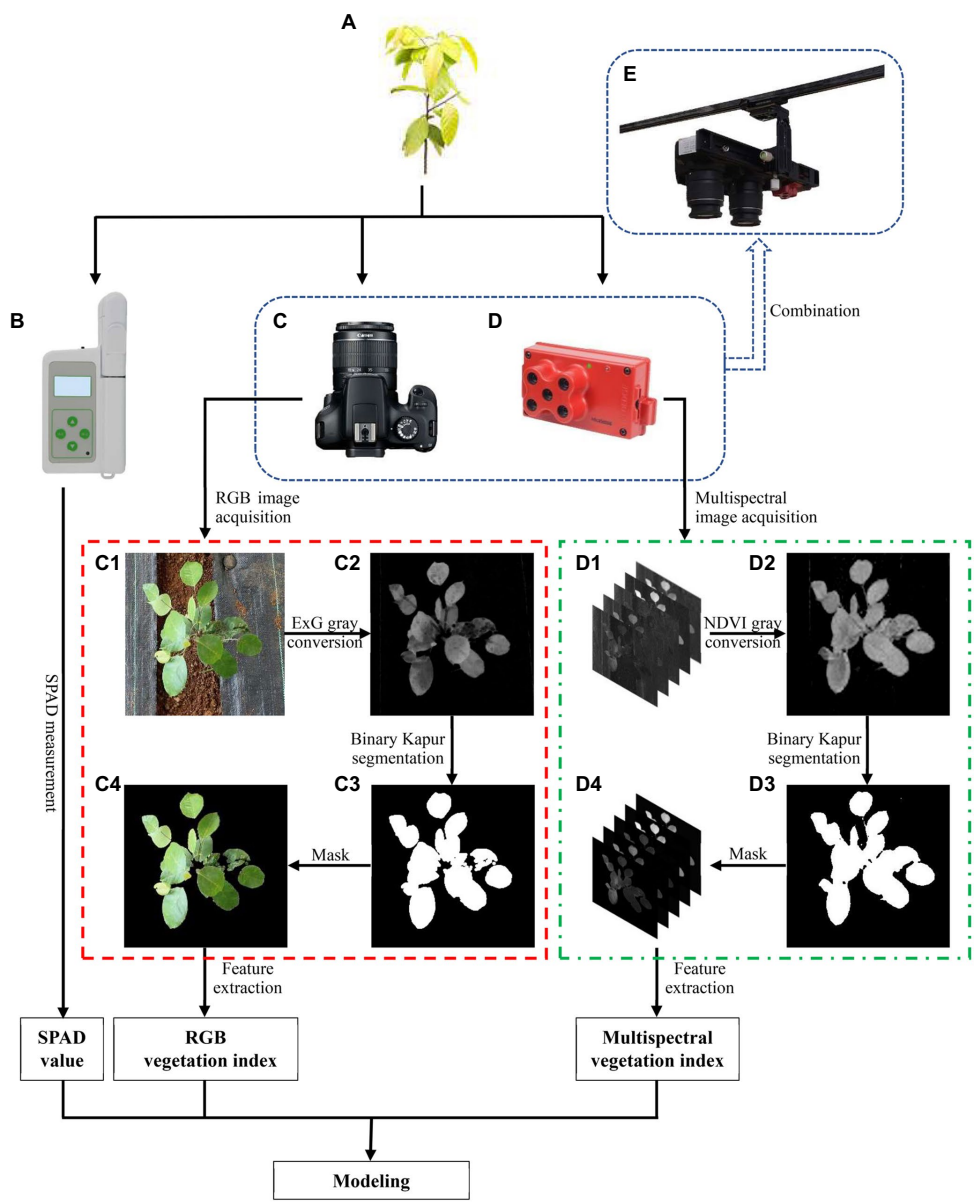
Flow chart of data measurement and processing. **(A)** *Hopea hainanensis* seedling. **(B)** Portable plant nutrition meter (TYS-4N). **(C)** Digital camera (Cannon EOS 4000D). **(D)** MS camera (MicaSense Edge 3). **(E)** Combination device of digital camera and MS camera. (C1) Region of interest extracted from RGB image. (C2) Gray image based on ExG conversion. (C3) Binary image after Kapur segmentation. (C4) Mask result of C3–C1. (D1) Region of interest extracted from MS image. (D2) Gray image based on NDVI conversion. (D3) Binary image after Kapur segmentation. (D4) Mask result of D3–D1.

Figure 2 shows the flow of data acquisition and processing. For RGB images, the ExG index was used to calculate the separate single-channel images to convert RGB images to gray images. Then, the gray images were segmented by the maximum entropy threshold (Kapur) method to obtain binary images of the plants and background. We used mask technology to remove the backgrounds of the original RGB images by applying binary images.

There are differences in plant spectral information between RGB images and the corresponding red, green, and blue band images in MS images because the MS images only retain the narrow spectral information obtained by the narrowband spectral sensors. We achieved vegetation segmentation through a similar process; however, the VI used for gray conversion to highlight the plant parts in images was changed from ExG to NDVI, which was proven to be suitable for narrowband MS images in a previous study (Jawak et al., 2019).

#### VI extraction

The RGB images of the seedling canopy were separated into three grayscale images corresponding to the individual channels, and then the average DN values of the plant parts in the images of the individual R, G, and B channels were calculated. And the DN values were corrected based on white plate correction to eliminate the influence of scene brightness difference. According to the correction values, 10 RGB VIs were acquired (Table 2). These VIs were used to show the changes in visible light spectral information between seedling canopies with different chlorophyll contents.

**Table 2:** The information of RGB VIs.

Index	Abbreviation	Formula	References
Excess green	ExG	2 * G − R − B	Woebbecke et al. (1995)
Excess green minus excess red	ExGR	ExG − (1.4 * R − G)	Meyer and Neto (2008)
Normalized green–red difference index	NGRDI	(G − R)/(G + R)	Woebbecke et al. (1993)
Normalized blue–red difference index	NGBDI	(G − B)/(G + B)	Woebbecke et al. (1993)
Red green ratio index	RGRI	R/G	Gamon and Surfus (1999)
Green blue ratio index	GBRI	B/G	Sellaro et al. (2010)
Color index of vegetation extraction	CIVE	0.441 * R − 0.811 * G + 0.385 * B + 18.78745	Kataoka et al. (2003)
Vegetative index	VEG	G/(R^A^ * B^(1−A)^), a = 0.667	Hague et al. (2006)
Red green blue vegetation indices	RGBVI	(G^2^ – B * R^2^)/(G^2^ + B*R^2^)	Possoch et al. (2016)
Modified green red vegetation indices	MGRVI	(G^2^ − R^2^)/(G^2^ + R^2^)	Bendig et al. (2015)

The MS images consisted of five spectral bands that increase the information of near-infrared and red-edge bands compared with RGB images. These two bands were proven to be sensitive to the nitrogen content in previous studies (Huang et al., 2017; Prey et al., 2020), which is closely related to the chlorophyll content. Therefore, we calculated the average DN correction values of five images after white plate correction to obtain 10 MS VIs that were completely different from RGB VIs (Table 3) to make full use of these spectral bands.

**Table 3:** The information of MS VIs.

Index	Abbreviation	Formula	References
Normalized difference vegetation indices	NDVI	(NIR − Red)/(NIR + Red)	Rouse et al. (1974)
Ratio vegetation indices	RVI	NIR/Red	Pearson and Miller (1972)
Difference vegetation indices	DVI	NIR − Red	Liu and Huete (1995)
Enhanced vegetation indices	EVI	2.5 * (NIR − Red)/(NIR + 6 * Red − 7.5 * Blue + 1)	Huete et al. (1997)
Renormalized difference vegetation indices	RDVI	(NDVI/DVI)^0.5^	Liu and Huete (1995)
Red-edge vegetation indices	REVI	NIR/RE − 1	Gitelson et al. (2006)
Normalized difference red-edge index	NDRE	(NIR – RE)/(NIR + RE)	Siegmann et al. (2013)
Red-edge ratio vegetation indices	RERVI	NIR/RE	Prey et al. (2020)
Red-edge difference vegetation indices	REDVI	NIR − RE	Cao et al. (2013)
Simplified canopy chlorophyll content index	sCCCI	NDRE/NDVI	Fitzgerald et al. (2006)

### Data analysis and modeling

#### Variance inflation factor and lasso selection

In multivariate statistical analysis of VIs and SPAD values, the possible multicollinearity between VIs will affect the stability of the estimated values of model parameters. Variance inflation factor (VIF) statistics are usually used to detect the presence of collinearity in multiple linear models (Kroll and Song, 2013). By regressing each VI as the explanatory variable with other VIs, the VIF was calculated according to the following formula:


(1) $$\mathrm{VIF}=\frac{1}{1-R^{2}}$$

where *R*^2^ represents the coefficient of determination of the model. If VIF < 10, there is no multicollinearity between explanatory variables. If 10 ≤ VIF ≤ 20, there is a certain amount of autocorrelation between explanatory variables. If VIF > 20, there is serious multicollinearity between explanatory variables.

Lasso is a variable selection technique proposed by Tibshirani (1996), which is referred to as the least absolute selection and shrinkage operator. In this algorithm, a penalty function (L1 penalty) is constructed to compress the model coefficients, and some coefficients with small absolute values are decreased to 0 to achieve the effect of variable selection and solve the problem of multicollinearity (Liu et al., 2021). When the number of explanatory variables is i and the sample size is m, the original explanatory variables are standardized to $X=\left(x_{i1},x_{i2},\ldots ,x_{im}\right)$ with a mean of 0 and variance of 1 by linear transformation; these variables are used as the input variables of the regression model. Lasso estimates for the regression model are as follows.


(2) $${\hat{\beta }}_{\mathrm{Lasso}}=\arg\min{\left\|y-\overset{p}{\underset{j=1}{\sum }}x_{j}\beta_{j}\right\|}^{2}+\lambda \overset{p}{\underset{j=1}{\sum }}\left|\beta_{j}\right|$$

where *y* represents the output variable, *λ* is a nonnegative regularization parameter, and *p* represents the number of explanatory variables. The *λ* was obtained by iterative calculation with the mean square error of model as the objective function.

#### Shade dummy variables and random effects

In this study, four shade levels were set: 0%, 25%, 50%, and 75% shade. The SPAD values of *H. hainanensis* seedlings were different under different shade conditions. The common approach to deal with categorical variables such as shade is to include them in the estimation model of SPAD as dummy variables (Li et al., 2020). Dummy variables was determined by the categorical variable (shading level) and were added to the model by coding the categorical variable with 0 and 1 to make the model more adaptable to the local characteristics. Assuming that the original model is the ordinary least squares regression (OLR) model, the corresponding dummy variable model has the following form:


(3) $$\mathrm{SPAD}=bx+\overset{n}{\underset{i=1}{\sum }}a_{i}z_{i}+\varepsilon$$

where *z_i_* is the dummy variable, *a_i_* is the corresponding specific or local parameter, *x* represents the quantitative explanatory variables such as VIs, *b* is the corresponding regression coefficient, and *ε* represents the error matrix. The regression coefficients were calculated by least square.

In addition to dummy variables, random effects can also be used as structural forms of categorical variables in the model, such as the canopy structure (Li et al., 2021), which can also reflect their ability to explain the response variables. In the linear mixed effect model (LMM), the quantitative variables are fixed effects, and categorical variables are random effects. Random effects are assumptions about the heterogeneity and randomness of data caused by the categorical variables. We assumed that the fixed effect (image features) parameters under different shade levels were random. The corresponding LMM had the following form:


(4) SPAD=bx+cτ+ε

where *τ* represents the matrix of the random effects (shade levels) and *c* represents the coefficient matrix of random effects. The *b* and *c* were calculated by restricted maximum likelihood estimation.

#### Random forests

Random forest (RF) is a nonlinear machine learning algorithm that performs classification or regression through the prediction of a set of decision trees (Breiman, 2001). The decision trees that are not related to each other generate classifier models for independent learning and prediction. The final output of RF is determined by all decision trees to overcome the overfitting problem of a single decision tree (Xie et al., 2022). The sample selection of each tree in RF is random sampling with the replacement of the original data set. If the number of sampling times is N, N different decision trees are generated. With respect to the classification problem, the output is the class with the largest voting probability among the classification results of all decision trees. With respect to the regression problem, the output is the average of the prediction results of all decision trees.

#### Support vector regression

Support vector regression (SVR) is a supervised learning method for dealing with regression problems based on the extension of the support vector machine (SVM) for dealing with classification problems (Cristianini and Shawe-Taylor, 2000). SVR has the advantages of a simple structure and rapid calculation. The core of SVR is to map the training samples in low-dimensional space to the high-dimensional space nonlinearly related to the original feature space through a transfer function and make the distribution of the new data set more suitable for the linear model (An et al., 2020). There are many options for transfer functions, such as polynomial functions, sigmoid functions, and RBFs. Due to the better fitting effect of RBF in the estimation of the leaf nitrogen concentration of wheat (Wang et al., 2017), RBF was selected as the transfer function of the SVR model used the 10-fold cross validation method to determine the optimal parameters in this study.

#### Model evaluation and validation

In the comparison of the fitting effect of the model, the coefficient of determination (*R*^2^), root mean square error (RMSE) and mean absolute percent error (MAPE) were used as the evaluation indices. As a positive index, the closer the value of *R*^2^ is to 1, the better the fit. As negative indices, the smaller the RMSE and MAPE values, the smaller the estimation error. The samples were divided into modeling samples with a sample size of 150 and test samples with a sample size of 50 by random sampling based on a 3:1 ratio to test the application ability of the model.

## Results

### Effect of shade levels on SPAD values

**Figure 3:**
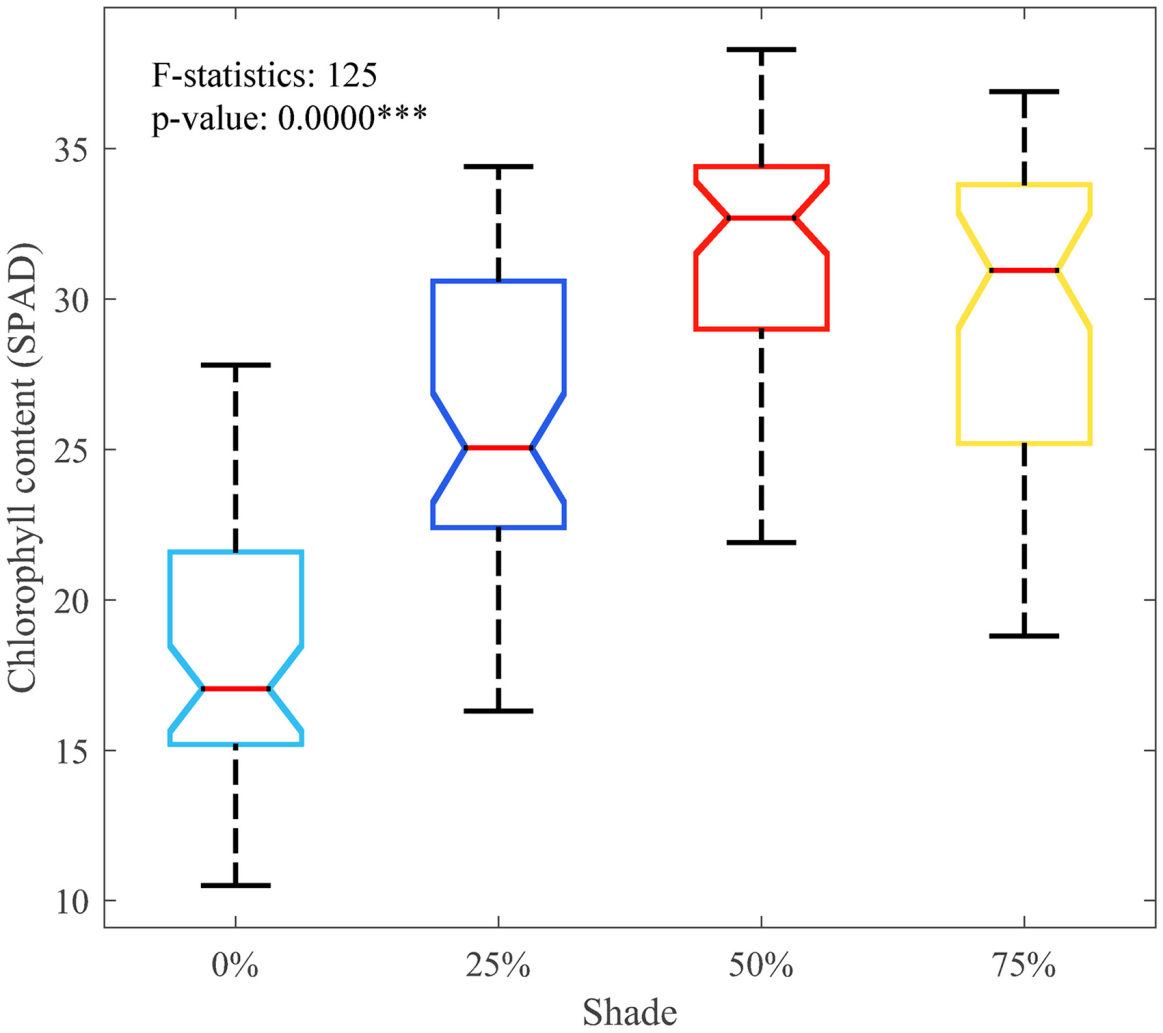
Chlorophyll content (SPAD) data distribution of seedlings under different shade levels. ***p < 0.001.

Under the four shade levels, the average SPAD values of the seedlings were 18.39, 25.76, and 31.81 when the shade degrees were 0%, 25%, and 50%, respectively. The SPAD values were positively correlated with the degree of shading. However, when the shade increased to 75%, the average SPAD value decreased to 29.26. The SPAD distribution of seedlings under different shade levels is shown in Figure 3. The calculated *F*-statistic was 125, *p*-value < 0.01. Therefore, the SPAD values under different shade levels showed significant differences within the 95% confidence interval. For the growth of 2-year-old *H. hainanensis* seedlings, 50% shade is appropriate, which can increase the chlorophyll content of the seedlings compared with no shade (0%), low shade (25%) and excessive shade (75%).

### RGB and MS VI selection

In the analysis of the modeling samples, the Pearson correlation coefficient (*R*) between the SPAD values and VIs from the RGB and MS images and the *p*-values representing significance were calculated. In addition, the VIFs of each VI were also calculated. The statistical results of the RGB VIs are shown in Table 4. The analysis results showed that among the 10 RGB VIs, 8 VIs had a significant correlation with SPAD in the 95% confidence interval; VEG and RGBVI did not exhibit a significant correlation. The Pearson correlation coefficients of all RGB VIs and SPAD values ranked from high to low as RGRI, MGRVI, NGRDI, ExGR, NGBDI, GBRI, CIVE, ExG, VEG, and RGBVI. RGRI had the highest correlation with the SPAD values.

**Table 4:** VIF and correlation with SPAD in RGB VIs.

RGB VIs	Mean ± standard deviation	*R*	*p*-value	VIF
ExG	0.69 ± 0.08	0.1859	0.0228^**^	3.94E+04
ExGR	0.57 ± 0.10	0.4106	0.0000^***^	820.7527
NGRDI	0.21 ± 0.04	0.4256	0.0000^***^	431.3603
NGBDI	0.50 ± 0.08	−0.2469	0.0023^***^	305.4540
RGRI	1.01 ± 0.06	−0.4371	0.0000^***^	65.9226
GBRI	0.91 ± 0.10	0.2322	0.0042^***^	15.0777
CIVE	18.70 ± 0.03	−0.2213	0.0065^***^	4.85E+04
VEG	1.57 ± 0.07	0.1162	0.1568	19.7474
RGBVI	0.52 ± 0.06	−0.0781	0.3420	63.4681
MGRVI	0.41 ± 0.07	0.4297	0.0000^***^	580.1231

** *p* < 0.01;

*** *p* < 0.001.

The statistical results of the MS VIs are shown in Table 5. The F test indicated that the correlation between each MS VI we calculated and the SPAD value was significant within the 95% confidence interval. The order of the correlation coefficients from high to low is as follows: NDRE, REDVI, sCCCI, DVI, RDVI, NDVI, RVI, EVI, REVI, and RERVI. NDRE had the highest correlation with the SPAD value.

**Table 5:** VIF and correlation with SPAD in MS VIs.

MS VIs	Mean ± standard deviation	*R*	*p*-value	VIF
NDVI	0.96 ± 0.07	0.3458	0.0000^***^	1.6832
RVI	5.07 ± 0.76	0.3045	0.0002^***^	0.1818
DVI	0.64 ± 0.11	0.3551	0.0000^***^	1.7496
EVI	1.28 ± 0.17	0.2617	0.0012^***^	0.0373
RDVI	2.01 ± 0.16	−0.3511	0.0000^***^	0.9178
REVI	1.08 ± 0.23	0.2418	0.0029^***^	−1.19E+13
NDRE	0.89 ± 0.13	0.3738	0.0000^***^	13.5474
RERVI	2.08 ± 0.23	0.2418	0.0029^***^	−1.19E+13
REDVI	0.32 ± 0.09	0.3696	0.0000^***^	0.1521
sCCCI	0.92 ± 0.11	0.3579	0.0000^***^	4.6747

*** *p* < 0.001.

A comparison indicated that the performance changes in RGB VIs were greater than that in MS VIs. For example, the absolute *R* values of ExGR, NGRDI, RGRI, and MGRVI were >0.4, i.e., higher than that of any MS VI, while the performances of other VIs were worsethan those of MS VIs. In contrast, there was little difference in the correlation analysis results of the SPAD value and MS VIs.

**Figure 4:**
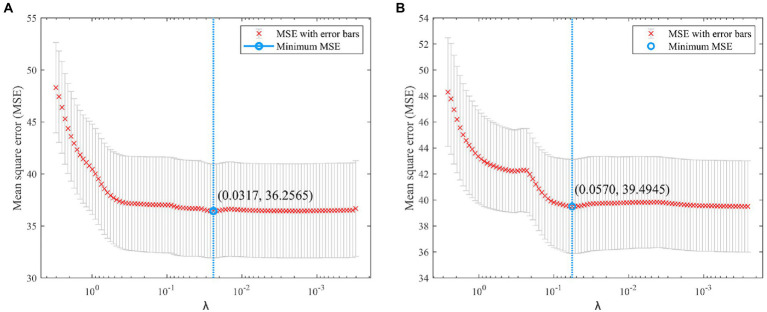
*λ*-value iterative process of Lasso algorithm with error bar. **(A)** RGB-based Lasso. **(B)** MS-based Lasso.

In terms of multicollinearity analysis, the VIFs corresponding to most VIs were >10, which indicates serious multicollinearity. The VEG and RGBVI with low correlation with the SPAD values were eliminated, and the remaining VIs and SPAD values were used as input variables of the Lasso model for feature screening. Figure 4 shows the *λ* parameter iteration process of running the Lasso algorithm with the mean square error (MSE) as the objective function in the selection process of RGB and MS VIs. The best *λ* value of the Lasso model based on the RGB VIs was 0.0317, and the value of the Lasso model based on MS VIs was 0.0570.

Table 6 shows the selection results of the Lasso model. Among the RGB VIs, ExG, ExGR, NGRDI, and NGBDI were retained. The complex correlation coefficient of the Lasso regression model constructed by four RGB VIs and the SPAD value was 0.5396, which is higher than that of any single VI. The overall *p*-value of the model was <0.01, indicating that the regression equation was significant. Among MS VIs, NDVI, EVI, RDVI, REVI and REDVI were retained. The complex correlation coefficient of the Lasso model was 0.4835, and its *p*-value was also <0.01. Most importantly, the VIFs of the retained VIs were greatly reduced to <5 through the screening of the Lasso algorithm. This means that multicollinearity was effectively eliminated.

**Table 6:** Selection results of VIs using Lasso algorithm.

VIs		*R*	*p*-value	VIF
RGB VIs	ExG	0.5396	0.0000^***^	0.2563
	ExGR			0.6378
	NGRDI			0.3589
	NGBDI			0.0362
MS VIs	NDVI	0.4835	0.0000^***^	0.4331
	EVI			0.0305
	RDVI			0.3752
	REVI			0.0986
	REDVI			0.1148

*** *p* < 0.001.

### SPAD estimation model

#### Modeling of VIs and the SPAD value

**Figure 5:**
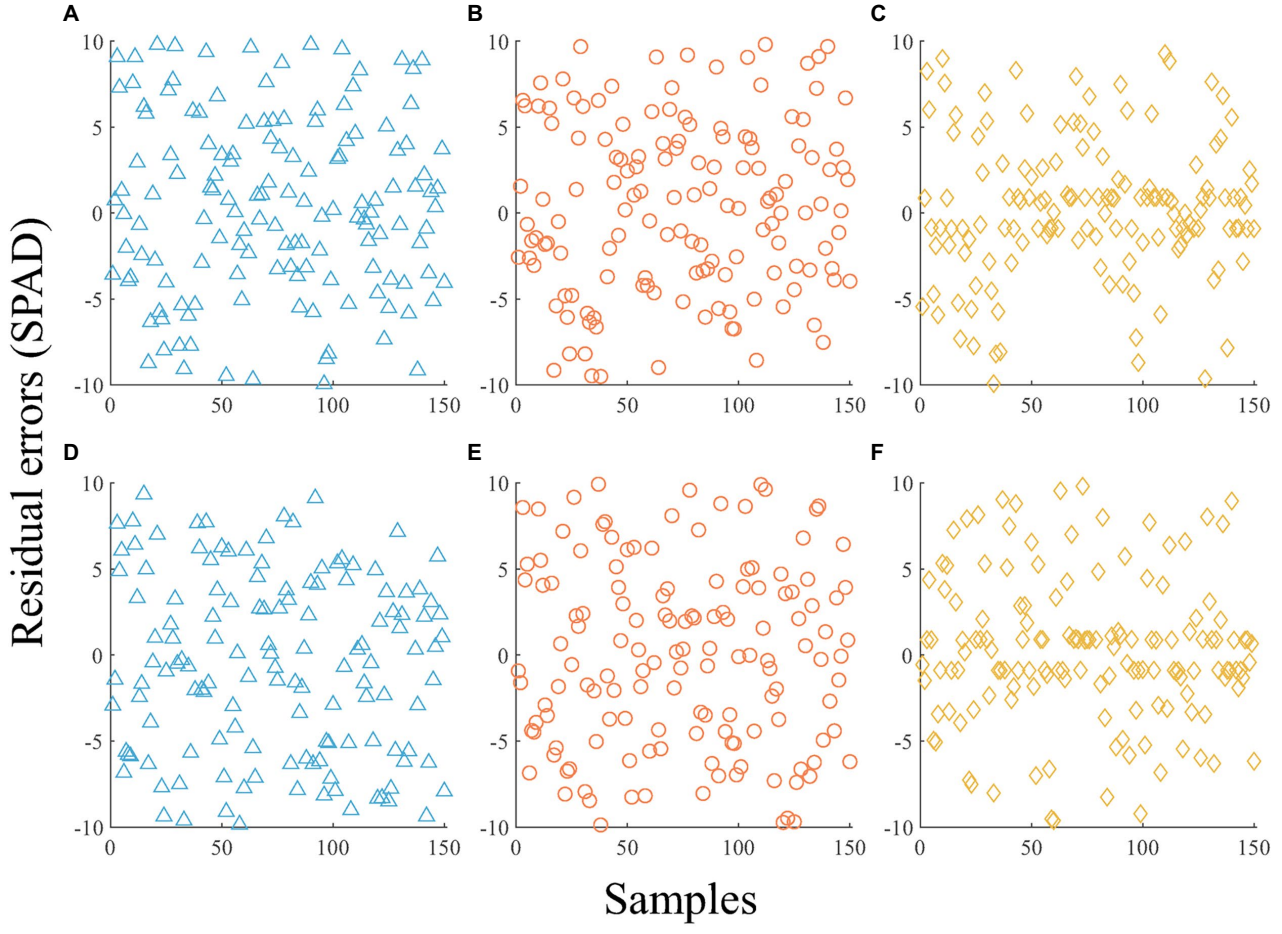
Estimated residuals of modeling samples using different images and algorithms without shade variables. **(A)** RGB-based OLR. **(B)** RGB-based RF. **(C)** RGB-based SVR. **(D)** MS-based OLR. **(E)** MS-based RF. **(F)** MS-based SVR.

The selected RGB and MS VIs were used as explanatory variables and the SPAD value was used as the response variable in the construction of the OLR, RF, and SVR models. The residual errors calculated by all models are shown in Figure 5. With the *R*^2^, RMSE, and MAPE as evaluation indices, the models constructed with different image data and different algorithms were compared, and the results are shown in Table 7.

**Table 7:** Evaluation of the OLR, RF, and SVR models without shade variables.

Model	RGB VIs			MS VIs		
	*R* ^2^	RMSE (SPAD)	MAPE	*R* ^2^	RMSE (SPAD)	MAPE
OLR	0.2912	5.8242	20.97%	0.2337	6.0558	22.57%
RF	0.4108	5.4523	20.18%	0.3007	5.8475	21.99%
SVR	0.4899	4.9465	15.56%	0.5310	4.7830	15.35%

The residual errors of the OLR model were higher than those of the RF and SVR models, and the fitting effect was poor for both RGB VIs and MS VIs. According to Table 7, the algorithm with the best fitting effect was SVR. The *R*^2^ of RGB-based SVR was 0.4899, which is 68.23% and 19.26% higher than that of OLR and RF, the RMSE was 4.9465, which is 15.07% and 9.28% lower than that of OLR and RF, and the MAPE was 15.56%, which is 25.80% and 23.89% lower than that of OLR and RF. The *R*^2^ of MS-based SVR was 0.5310, which is 127.21% and 76.59% higher than that of OLR and RF, the RMSE was 4.7830, which is 21.02% and 18.20% lower than that of OLR and RF, and the MAPE was 15.35%, which is 31.99% and 30.20% lower than that of OLR and RF. The results show that the SVR algorithm fit the relationship between VIs and the SPAD value better than OLR and RF, but the estimation accuracy was still not high because the growth difference of seedlings under different shade levels was not considered.

#### Modeling of VIs, shade, and the SPAD value

**Figure 6:**
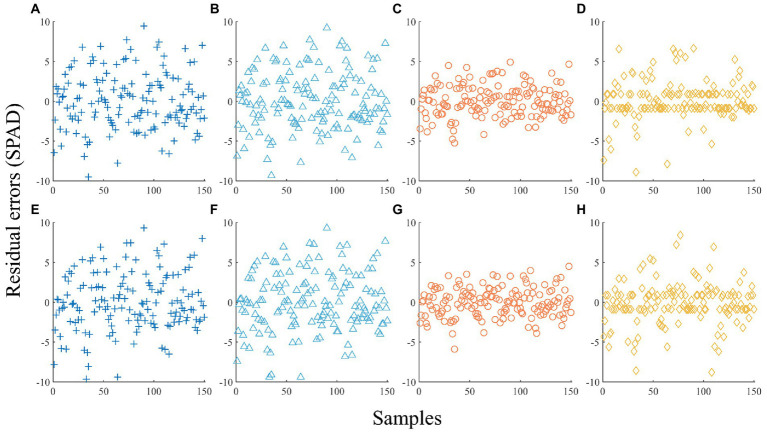
Estimated residuals of modeling samples using different images and algorithms with shade variables. **(A)** RGB-based LMM. **(B)** RGB-based OLR. **(C)** RGB-based RF. **(D)** RGB-based SVR. **(E)** MS-based LMM. **(F)** MS-based OLR. **(G)** MS-based RF. **(H)** MS-based SVR.

The shade levels were added to the modeling process. In LMM, the SPAD values were estimated with the shade levels as the random effects and the VIs of the RGB or MS images as the fixed effects. In the OLR, RF, and SVR algorithms, dummy variables reflecting the shade levels were designed, together with VIs, and were used as explanatory variables to build an estimation model with the SPAD value as the response variable. The estimated residual errors of the LMM, OLR, RF, and SVR models separately constructed based on the RGB and MS images for the modeling samples are shown in Figure 6. The *R*^2^, RMSE, and MAPE were calculated for all models (Table 8) to facilitate quantitative comparison.

**Table 8:** Evaluation of the LMM, OLR, RF, and SVR models with shade variables.

Model	RGB VIs			MS VIs		
	*R* ^2^	RMSE (SPAD)	MAPE	*R* ^2^	RMSE (SPAD)	MAPE
LMM	0.7231	3.6410	12.24%	0.7278	3.6099	11.90%
OLR	0.7138	3.7008	12.39%	0.7168	3.6817	12.21%
RF	0.9273	1.9844	6.73%	0.9389	1.8109	6.10%
SVR	0.8651	2.6019	7.53%	0.8595	2.6364	8.16%

Figure 6 indicates that the fitting effects of RF and SVR with the shade variables were significantly better than those of the models without the shade variables (Figure 5) irrespective of RGB or MS images. According to the *R*^2^, RMSE, and MAPE, the order of RGB-based and MS-based model accuracy was RF > SVR > LMM > OLR. For the optimal RF model, *R*^2^ based on RGB and MS VIs reached 0.9273 and 0.9389, respectively. The *R*^2^ value of the SVR model with the next highest goodness of fit reached 0.8651 and 0.8595, while the *R*^2^ values of the LMM and OLR models were lower than 0.8. A comparison of the interpretation ability of RGB and MS VIs under the same algorithm indicated that MS VIs were superior to RGB VIs based on the RF, OLR, and LMM algorithms, while RGB VIs achieved better performance in SVR.

#### Model validation

Both RF and SVR performed better than LMM or OLR in the construction of the SPAD estimation models with or without different shade levels. The structures of the RF and SVR models constructed by the modeling samples were tested with test samples to determine the generalization ability of the models and to determine whether there was an overfitting phenomenon. In the test results (Table 9), the models considering the shade levels still had a significantly stronger ability to estimate the SPAD values. The *R*^2^ values of the RF and SVR models without the shade variables were lower than 0.4, which is far lower than the values of the models with dummy variables constructed for different shade levels. This indicates that it is necessary to consider the influence of the shade level in the process of modeling to determine the SPAD value.

**Table 9:** Evaluation of RF and SVR models for testing samples.

Model		RGB VIs			MS VIs		
		*R* ^2^	RMSE (SPAD)	MAPE	*R* ^2^	RMSE (SPAD)	MAPE
Without shade variables	RF	0.3774	5.8646	19.30%	0.3173	5.8545	19.11%
	SVR	0.3059	6.0529	19.18%	0.1300	6.5385	20.61%
With shade variables	RF	0.7854	3.1582	9.38%	0.8013	2.9616	9.04%
	SVR	0.5769	4.3624	13.49%	0.6771	3.7816	10.86%

With respect to the models with shade variables, RF had better estimation accuracy than SVR. Although the performance for the test samples was lower than that for the modeling samples, the *R*^2^ of RF remained at ~0.8, which indicates that RF did not overfit the modeling samples. However, the estimation accuracy of SVR decreased significantly. For example, the *R*^2^ decreased from 0.8651 to 0.5769 for the modeling samples when the SPAD value was estimated using RGB VIs, i.e., a decrease of 33.31%. Therefore, RF was more suitable for estimating the SPAD value of *H. hainanensis* seedlings based on RGB or MS VIs. In addition, based on a comparison of the performance of RGB and MS VIs, the MS-based RF had a higher *R*^2^ (0.8013), lower RMSE (2.9616 SPAD value) and lower MAPE (9.04%). Accordingly, the MS VIs had a greater ability to explain the SPAD data.

## Discussion

### Differences in SPAD values under different shade levels

Shade can change the light conditions of the plant growth environment, thus affecting photosynthesis and further causing changes in the chlorophyll content in plant leaves. Different plants have different adaptations to shade due to their genetic characteristics. A study showed that in *Tetrastigma hemsleyanum*, chlorophyll a, chlorophyll b, and the total chlorophyll content increased and the chlorophyll a/b values decreased with increasing shade (Dai et al., 2009). Another study showed that under different culture conditions, SPAD values and chlorophyll contents were higher beneath a cover than under open culture (Sano et al., 2018). To explore the effect of shade on *H. hainanensis* seedlings, four shade levels were set up in this study, and the SPAD values representing the chlorophyll content of 50 *H. hainanensis* seedlings were measured at each level. A significant correlation between the shade level and the SPAD value was indicated by the *F* test. The results showed that 50% shade was beneficial for chlorophyll accumulation in *H. hainanensis* seedlings. This finding is similar to the results of previous experiments conducted by Yang and Chen (2017), who showed that shade was beneficial to the growth of *H. hainanensis* seedlings. In contrast, the results of our study supplement the mathematical analysis of the phenomenon and provide quantitative guidance.

We consider that as a tropical understory plant, *H. hainanensis* has developed the genetic characteristics of shade tolerance under the influence of the high canopy density of tropical rainforests. Therefore, appropriate shade at the seedling stage plays a positive role in seedling growth and development, and ~50% shade is a suitable choice. In addition, in the comparison of estimation models, the fitting effect of the models with shade variables was greatly improved because there were differences in the changes of SPAD values under different shade levels (Figure 3), and dummy variables and random effects improved the local interpretation ability of the model by adjusting the model parameters at different levels. The improvement of model accuracy further shows the difference in the physiological characteristics of *H. hainanensis* seedlings under different shade levels and the necessity of considering shade conditions in estimating the SPAD value. Shade control is a common and effective management measure for *H. hainanensis* seedlings, so the analysis of shading conditions in this study is of great practical significance, which can improve the accuracy and flexibility of SPAD estimation under different shade conditions.

### Selection of VIs for estimating SPAD values

VIs are composed of different spectral bands that can reflect the growth status of plants. By comparing the difference in the SPAD estimation performance of individual VIs, the best VI that can reflect the SPAD value can be obtained. For example, in a study estimating the chlorophyll content of potato leaves, RVI was considered to be the best monitoring index (Kooistra and Clevers, 2016). When the estimation accuracy of a single VI is sufficient, its application is more simplified than that of multiple VIs. However, the prediction method of multiple VIs can improve the accuracy of the model to some extent. Zhou et al. (2018) found that high model accuracy was obtained when monitoring the leaf nitrogen concentration in rice crops based on multiple VIs.

In this study, among RGB VIs, RGRI had the highest correlation with chlorophyll when the individual VI was used to analyze the SPAD data, and the correlations between VEG and RGBVI and the chlorophyll content were not significant. Among MS VIs, NDRE exhibited the best performance, and the correlation was higher than the commonly used NDVI and other VIs. This is similar to the experimental results obtained by Carneiro et al. (2020), who compared the performance of NDVI and NDRE when monitoring soybean variability. The differences in spectral characteristics between plants lead to differences in the applicability of VIs.

Although the correlation between most VIs and the SPAD values of the *H. hainanensis* seedlings was significant, the interpretation of an individual VI was insufficient (maximum absolute *R* = 0.4371). Multicollinearity usually exists when multiple VIs are used as explanatory variables, which was also verified by the excessively high VIF values we calculated. After the RGB and MS VIs were screened by the Lasso algorithm, the VIF values were all reduced to <10, indicating that the screened VIs had no multicollinearity. It is noteworthy that among the retained VIs, A and B were not retained and were highly correlated in the correlation analysis between the individual VI and the SPAD value. This is because when a variable has strong collinearity with other explanatory variables, the Lasso algorithm will remove one of the variables on the premise of considering the model accuracy (MSE; Huang, 2003). Compared with the method of variable screening only according to the correlation, in this way, the overall accuracy of the model is more strongly considered, which is conducive to the simultaneous analysis of multiple sources of information.

### Appropriate SPAD estimation models

Suitable mathematical models quantitatively reflect the relationship between VIs and the SPAD value with a stable model structure and parameters. In previous research, both the traditional algorithm of fitting model parameters (Peng et al., 2018) and the machine learning algorithm (Peng et al., 2021), which has developed rapidly in recent years, have been applied in nutrient estimation. It is meaningful to explore which algorithm is most suitable for monitoring the SPAD value of *H. hainanensis* seedlings. In this research, the performances of traditional models (LMM and OLR) and machine learning models (RF and SVR) in fitting the SPAD values of *H. hainanensis* seedlings were compared. Regardless of whether the shade variable was considered, the fitting effects of RF and SVR were better than those of the traditional algorithms for the two images. It is worth noting that reasonable use of machine learning algorithms can indeed obtain higher estimation accuracy than that obtained by traditional algorithms, but due to the more complex parameters of these algorithms, it is also easy to produce overfitting problems. To avoid these problems, the division of modeling and test samples is a common and effective method to test model overfitting. After sample verification, it was found that the SVR model did cause overfitting, while the performance of RF was more stable. Because RF is composed of multiple decision trees that allow independent prediction (Heenkenda et al., 2015), it has a huge advantage in preventing overfitting. Therefore, RF was the most suitable algorithm for monitoring the SPAD value of *H. hainanensis* seedlings compared with traditional algorithms (OLR and LMM) and SVR.

### Comparative performance of RGB and MS VIs

Due to the low cost of digital cameras and narrowband MS cameras, the potential of RGB and MS images to estimate the plant nutrient status is worth exploring. RGB images have higher resolution but less spectral bands than MS images, while the MS images contain more bands, including the near-infrared and red-edge bands sensitive to chlorophyll content, but lower resolution than RGB images. Comparing the performance of two images can provide a reference for selecting the most appropriate image data source. A study reported that the RGB index was more robust than MS index in estimating the maize grain yield (Gracia-Romero et al., 2017). Another study showed the superiority of the MS index for the nitrogen concentration and chlorophyll-a content of soybean (Maimaitijiang et al., 2017). After analyzing the SPAD values of *H. hainanensis* seedling leaves with a single VI, we obtained similar results to those reported by Gracia-Romero et al. (2017). The correlation between ExGR, NGRDI, RGRI, and MGRVI (RGB VIs) and the SPAD value was higher than that of any MS VI. However, based on the multivariate model, the research results are similar to those for wheat reported by Maimaitijiang et al. (2017). The MS VIs had better performance than the RGB VIs when fitting data using the LMM, OLR, and RF algorithms. Taking the calculation results of the RF algorithm with the highest accuracy as an example, we took the selected RGB VIs and MS VIs as the input variables of the RF model, and the output results showed that the RGB and MS VIs adequately estimated the SPAD values of *H. hainanensis* seedlings. However, in the modeling samples, the *R*^2^ of the MS-based model was higher than that of RGB VIs, and the RMSE and MAPE were lower, similar to the test samples. The MS VIs had a greater ability to interpret the SPAD data. Therefore, the MS images are worthy of consideration as image data sources to estimate the SPAD values of *H. hainanensis* seedlings when multiple VIs are applied. Moreover, the combination of RGB and MS indices has achieved good results in rice yield prediction (Wan et al., 2020). It is also worthy of our further exploration in the *H. hainanensis* monitoring in the future.

### Limitations and prospects

The image-based estimation method is a nondestructive alternative to the chemical measurement of plant physiological indices. Combined with the self-learning machine method, it has the advantages of being fast and quantitative. Because of the scarcity of endangered species, destructive chemical measurements are difficult to apply in large numbers, whereas nondestructive monitoring methods can be used to observe the changes in plant images to indirectly capture the physiological and growth changes of endangered plants over time. For the estimation algorithms, the best estimation method in this experiment was the RF algorithm based on MS images and dummy variables. The *R*^2^ values of the models for the modeling and test samples were 0.9389 and 0.8013, respectively, which proves the feasibility of the method that has advantages over previous studies (Lu et al., 2019). In consideration of the power of deep learning in learning ability, in future work, we will obtain more data through continuous observation to explore the application of deep learning in SPAD estimation. Moreover, it cannot be ignored that the method proposed in this paper was the monitoring of a single seedling based on the ultralow altitude platform (2 m), which is designed according to the demand of the number of samples for high-resolution images. The estimation effect for larger sites and taller trees needs to be explored in future work. For example, the aerial imaging platform may be a good choice. In recent years, the method by which UAVs carry spectral imaging sensors has received continuous attention (Kefauver et al., 2017). After appropriate adjustment, the method proposed in this study can be applied to UAV images to make the monitoring range more extensive. In addition, the spectral characteristics of leaves of different plants vary, so more plants should be considered in subsequent studies to explore the applicability of this method for endangered plants.

## Conclusion

In this study, we proposed a lossless and low-cost image estimation method for the chlorophyll content of *H. hainanensis* seedlings under different shade levels, analyzed the estimation effect of traditional algorithms and machine learning algorithms, and compared the performance of RGB VIs and MS VIs. The results show that shade had a significant effect on the chlorophyll content of *H. hainanensis* seedlings, and the average chlorophyll content of seedlings under 50% shade was the highest. Based on the Pearson correlation coefficient as an indicator, RGRI had the highest correlation with the SPAD value among RGB VIs, while NDRE had the highest correlation with the SPAD value among MS VIs. After Lasso screening, ExG, ExGR, NGRDI, and NGBDI (RGB VIs) and NDVI, EVI, RDVI, REVI, and REDVI (MS VIs) were retained, and multicollinearity was eliminated. The optimal model was the RF model based on MS images and the dummy variables constructed by shade levels. The *R*^2^ values calculated for the modeling and test samples were 0.9389 and 0.8013, respectively. Additionally, the MS images were more suitable as image data sources due to the higher estimation performance of MS VIs for the SPAD value of *H. hainanensis* seedlings in the analysis of multiple VIs. These results provide a feasible and specific scheme for the nondestructive monitoring of chlorophyll in *H. hainanensis* seedlings and facilitate accurate management in the cultivation process of *H. hainanensis*. Timely and low-cost nutrient monitoring is also conducive to the protection and cultivation of endangered species.

## Data availability statement

The raw data supporting the conclusions of this article will be made available by the authors, without undue reservation.

## Author contributions

YY performed the experiments, analyzed the data, and wrote the manuscript. XW designed the research and conducted the field measurements and the collection of samples. MS performed the experiments and processed images. PW analyzed the data. All authors contributed to the article and approved the submitted version.

## Funding

This research was funded by the National Natural Science Foundation of China (no. 32071761). We also acknowledge the support from the IFRIT of CAF.

## Conflict of interest

The authors declare that the research was conducted in the absence of any commercial or financial relationships that could be construed as a potential conflict of interest.

## Publisher’s note

All claims expressed in this article are solely those of the authors and do not necessarily represent those of their affiliated organizations, or those of the publisher, the editors and the reviewers. Any product that may be evaluated in this article, or claim that may be made by its manufacturer, is not guaranteed or endorsed by the publisher.
